# Understanding the patient voice for medicine development: Qualitative research of the patient journey in Alzheimer's Disease

**DOI:** 10.1002/alz70858_100775

**Published:** 2025-12-24

**Authors:** Chiko Ncube, Dave Pearce, Harjeet Dhillon, Joanna Culver, Robert Y. K. Lai

**Affiliations:** ^1^ GSK, London, United Kingdom

## Abstract

Significant disparities exist throughout the patient journey and ultimately in health outcomes across diverse patient populations with Alzheimer's Disease (AD). The aim of this qualitative market research was to better understand the experiences of a diverse group of patients living with AD, to support inclusive and patient‐centered approaches in medicine development. Patients diagnosed with mild cognitive impairment (MCI) and dementia due to AD (mild, moderate, or severe), and care partners of patients living with AD, were included in this research. Participants were interviewed one‐to‐one or as dyads, with follow‐up ethnographic tasks. The research included a diverse group of patients across geography, ethnicity, race and employment status. Data was thematically analysed. A total of 65 participants (29 patients and 36 care partners) from the United States (US; 15%), Canada (20%), China (17%), France (12%), Germany (17%), and Italy (18%) were included. The mean patient age was 65.7 years, with the reported stage of disease as MCI or mild dementia due to AD (40%) and moderate or severe dementia due to AD (60%). The analysis revealed an overall consistent patient journey including diagnosis, day‐to‐day living, care, and treatment experiences. Some variations were revealed in the lived experiences of different patient groups. Many patients from diverse ethnic/racial groups emphasized the importance of family involvement in care and treatment decisions. Black participants in the US expressed concerns with seeking treatment due to lack of representation in medicine development and the need for future medications to be explicitly trialled in participants from ethnic/racial minorities. Asian participants in China, the US and Canada reported major stigma associated with AD, which delayed them in seeking medical help. Across all countries, patients living in rural locations experienced increased financial and logistical burden in accessing healthcare. These findings underscore the need to better understand the unique experiences faced by diverse populations of patients when considering the process for AD medicine development. A patient‐centered approach could help increase trust and ultimately improve the patient experiences and health outcomes for diverse populations affected by AD.

